# Fluorapatite Glass-Ceramics in Dentistry: Synthesis, Properties, Forming Technology, Applications, Challenges, and Future Perspectives

**DOI:** 10.3390/ma18040804

**Published:** 2025-02-12

**Authors:** Chengli Li, Gaoqi Wang, Shouren Wang, Wei Shen, Yujun Zhang, Junfeng Kang, Zhen Xiao

**Affiliations:** 1 School of Mechanical Engineering, University of Jinan, Jinan 250022, China; lichengli7111@163.com (C.L.); me_wangsr@ujn.edu.cn (S.W.); sw158516133@163.com (W.S.); me_xiaoz@ujn.edu.cn (Z.X.); 2 Department of Prosthodontics, School and Hospital of Stomatology, Cheeloo College of Medicine, Shandong University & Shandong Key Laboratory of Oral Tissue Regeneration & Shandong Engineering Laboratory for Dental Materials and Oral Tissue Regeneration, Jinan 250012, China; 3 School of Materials Science and Engineering, University of Jinan, Jinan 250022, China; mse_kangjf@ujn.edu.cn

**Keywords:** fluorapatite glass-ceramics, synthetic methods, properties, forming technologies, dental application

## Abstract

Fluorapatite glass-ceramics (FGC) have been widely used in dental ceramics due to their excellent aesthetic properties and biocompatibility. In recent years, new synthesis methods, forming technologies, and the continuous optimization of performance attributes have driven the application of FGC in dental veneers, coatings, composites, and other restorations. This review summarizes the current research and applications of this material in the dental field and looks forward to its future optimization directions. The article focuses on five aspects: the development of preparation techniques for FGC; advances in their application in dental restoration shaping technologies; the performance advantages and limitations of these materials as dental materials; the current application status in veneers, coatings, composites, and other restorations; as well as the challenges in the current applications and prospects. In addition, additive manufacturing technology shows extremely broad application potential in FGC molding and applications. This review is hoped to provide strong guidance for the further application of FGC in the dental field, promoting the integration of related research and industry upgrades better to meet the needs of clinical practice and patients.

## 1. Introduction

**Figure 1:**
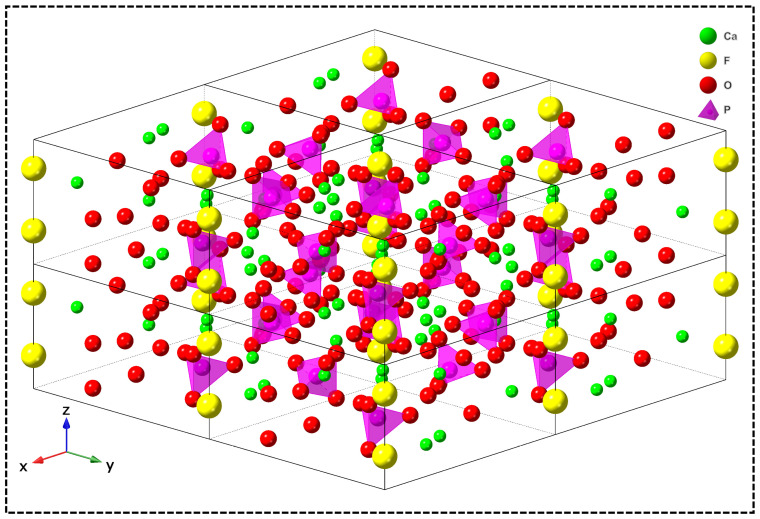
Crystal structure diagrams of FAp.

Dental materials encompass a diverse range of types, including composite resins, metals, and ceramics [1,2,3,4]. Among these, ceramic materials, such as zirconia (ZrO_2_), alumina (Al_2_O_3_), and glass-ceramic, play a pivotal role in dental treatments [5,6,7]. Fluorapatite (FAp; Ca_5_(PO_4_)_3_F or Ca_10_(PO_4_)_6_F_2_) glass-ceramics (FGC), composed of a glass matrix with acicular FAp crystals (Figure 1) dispersed within, have garnered significant attention in restorative dentistry. FGC exhibits a kinetically dominant growth along the crystallographic c-axis, with a pronounced expansion in this direction compared to the a-axis, which imparts superior microstructural and mechanical properties to the material [8,9]. Additionally, the material closely resembles natural enamel, and its stress intensity factor can be precisely determined through numerical methods, thereby offering valuable insights into the prevention and treatment of oral diseases [10]. Moreover, FGC material demonstrates exceptional biocompatibility, notable antibacterial properties, superior resistance to both acid and alkali corrosion, and favorable aesthetic outcomes. These characteristics position FGC as a promising dental restorative material with significant potential. A comprehensive investigation of its properties is essential to further optimize its application potential within the field of prosthodontics.

FGC is widely utilized in various dental applications, including veneers, coatings, filling materials, inlays, and crowns [11,12]. These materials are particularly effective in conservative treatments to mask unsightly shadows on substrates such as natural teeth, ZrO_2_, titanium (Ti), and its alloys. Furthermore, the application of FGC as coatings on dental restorations, such as crowns, bridges, and fixed brackets, significantly enhances both their aesthetic appearance and tribological compatibility. Ti and its alloys, commonly used in dental implants, have been shown to release metal ions due to corrosion, which can provoke immune responses in surrounding tissues, thereby increasing the risk of implant failure [13]. As a result, there has been growing interest in exploring non-metallic alternatives for dental implants, including ZrO_2_, Al_2_O_3_, and silicon nitride (Si_3_N_4_) [14]. While these biologically inert ceramic materials offer promising biocompatibility, challenges remain, such as the potential for ZrO_2_ to induce periodontitis and its relatively weak osseointegration properties [15,16]. However, coating implants with FGC has been demonstrated to enhance bone conductivity and promote osseointegration, addressing some of these limitations. Teeth primarily consist of an outer layer of enamel and an underlying layer of dentin [17]. The outer enamel surface is prone to damage under harsh conditions, including reciprocal motion, impact, and acid corrosion, leading to fractures, cracks, or abrasion. Such damage exposes the dentin to the oral environment, often resulting in tooth sensitivity [18,19]. Due to their composition, which closely resembles that of enamel, FGC is frequently employed as a restorative material to repair damaged teeth, restoring both function and appearance.

Although FGC has been extensively studied, several challenges and fundamental scientific issues persist in their practical applications. To date, there is no comprehensive review article that synthesizes the existing research and provides an in-depth discussion on the application of FGC in dentistry.

**Figure 2:**
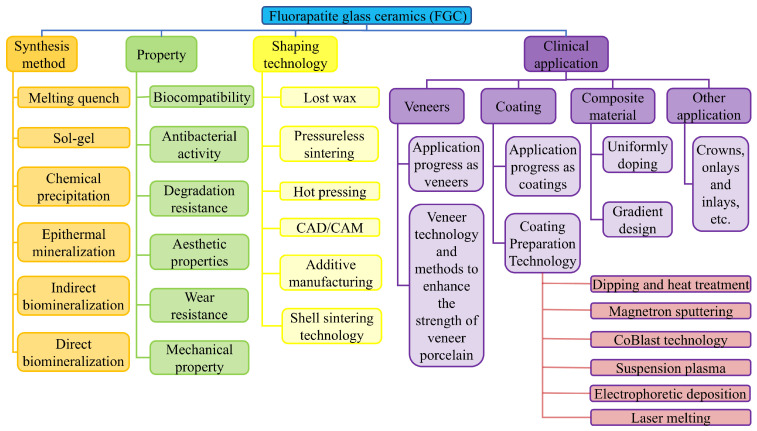
Synthesis, properties, forming, and application of FGC.

Therefore, this paper first reviews the research progress of FGC in the field of dentistry. Then, this paper focuses on the preparation method, forming process, performance characteristics, and limitations of FGC. Subsequently, the application status of FGC in dental restoration was summarized, including veneer, coating, and composite materials, and the related contents were reviewed in detail (Figure 2). Finally, this review highlights the challenges faced in the current clinical application and discusses the future development trends, aiming to provide valuable insights for the improvement and integration of FGC in dental practice. We anticipate that this review will help researchers gain a more comprehensive understanding of the current status of FGC as dental materials, thereby contributing to the future development of this field and ultimately enhancing the quality of life for dental patients.

## 2. Synthesis of FGC

**Figure 3:**
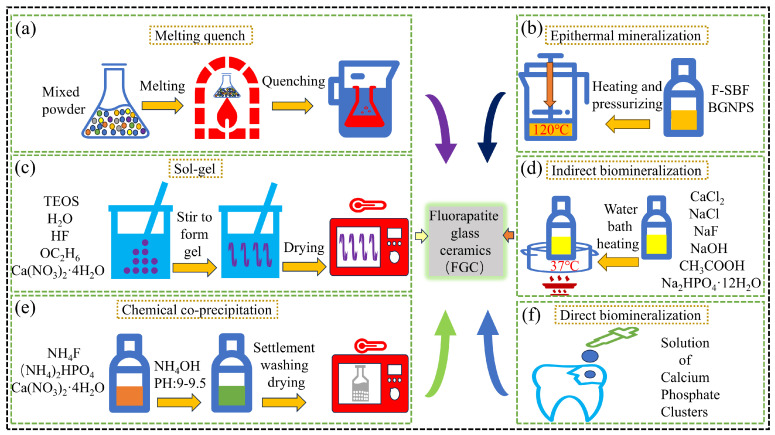
Methods for synthesizing FGC. (**a**) Melt-quenching; (**b**) epithermal mineralization; (**c**) sol–gel; (**d**) indirect biomineralization; (**e**) chemical co-precipitation method; (**f**) direct biomineralization.

In practical applications, FGC is not commonly found in nature and is typically synthesized through various methods. Among these, melt-quenching technology is the most widely used technique for the preparation of FGC. Other synthesis methods include the sol–gel process, chemical precipitation, and mineralization techniques. Each of these methods offers distinct advantages and limitations, and the selection of an appropriate synthesis route should be determined by the specific requirements of the intended application. A detailed flowchart of these synthesis methods is provided in Figure 3.

### 2.1. Melt-Quenching

The melt-quenching technique, one of the earliest methods employed for the preparation of glass-ceramics in suitable sizes, remains the preferred method for synthesizing the most practical glass-ceramics. The core principle of this technique involves melting pre-formulated raw material powders into a viscous liquid, followed by melt casting and rapid quenching [20]. As illustrated in Figure 3a, the synthesis process of FGC via melt quenching begins with the precise mixing of raw materials such as SiO_2_, Al_2_O_3_, Na_2_CO_3_, K_2_CO_3_, CaCO_3_, CaHPO_4_, and CaF_2_ in specific proportions. The mixture is then placed in a high-temperature resistor furnace and heated to approximately 1600 °C. Upon reaching the desired temperature, the molten mixture is rapidly quenched by immersion in deionized water, forming the base glass.

FGC produced through the melt-quenching technique has become the dominant method for preparing this material due to its excellent durability, absence of rupture or strain, ease of processing, and scalability. Despite these advantages, certain limitations of the process remain. Notably, the distribution of fluorapatite crystals within the amorphous base glass obtained after quenching tends to be highly heterogeneous. This necessitates subsequent mechanical operations such as breaking, grinding, and shaping, followed by secondary sintering to achieve the final product. Additionally, the high-temperature conditions required for the process can result in the evaporation of P_2_O_5_, potentially affecting the properties of the resulting glass-ceramics. This aspect of the melt-quenching technique requires further refinement to mitigate such issues. As summarized in Table 1, the raw material composition and content of FGC are prepared by melt-quenching.

### 2.2. Sol–Gel

In the synthesis of FGC, the sol–gel method begins with the preparation of the sol, which involves the dispersion of colloidal particles in a liquid medium. The specific procedure is outlined in Figure 3c. Initially, tetraethyl orthosilicate (TEOS), triethyl phosphate (TEP), calcium nitrate, distilled water, and hydrofluoric acid are mixed and stirred in precise proportions to initiate the hydrolysis and condensation reactions of TEOS. Following this, a drying step is performed, during which the sol is stirred until it transforms into a gel state. The gel is then subjected to further drying, and ultimately, it is calcined in an electric oven at temperatures ranging from 600 to 900 °C to yield FGC [33,34,35]. FGC synthesized via the sol–gel method exhibits smaller particle sizes compared to those produced by other techniques, with grain sizes ranging from 15 to 30 nm, resulting in a more uniform crystal distribution [36]. In comparison to FGC produced by melt-quenching, those derived from the sol–gel method tend to have higher porosity, which increases the specific surface area and enhances the material’s interaction with biological fluids. The smaller crystal size and larger specific surface area contribute to the bioactivity of the material [37,38]. However, the sol–gel method presents certain challenges in the synthesis of FGC. The shrinkage of the wet gel during drying may lead to the formation of cracks, which can adversely affect the mechanical properties of the final material. Furthermore, the primary limitations of this method include the high cost of raw materials and low production efficiency, which hinder its widespread industrial adoption.

### 2.3. Chemical Precipitation

As depicted in Figure 3e, the process of synthesizing FGC powder via the chemical precipitation method can be broadly divided into three stages. The first stage involves preparing an aqueous solution containing the initial components at the desired concentration. In the second stage, calcium nitrate (Ca(NO_3_)_2_·4H_2_O), ammonium dihydrogen phosphate ((NH_4_)_2_HPO_4_), and ammonium fluoride (NH_4_F) are combined to form the reaction solution. The final stage consists of adjusting the pH of the solution to 9–9.5 by the gradual addition of ammonium hydroxide (NH_4_OH), which induces the chemical reaction, leading to the precipitation of FGC. The resultant FGC is then recovered through washing, air drying, and grinding [39,40]. To produce higher-density FGC powders, the precipitated material is subjected to cold uniaxial pressing using a hydraulic press, followed by sintering at temperatures ranging from 1000 to 1250 °C for 6 h. The chemical precipitation method offers several advantages, including precise control over stoichiometry, lower synthesis temperatures, high purity, and good chemical homogeneity [41]. However, the method also has certain limitations, such as the need for the repeated washing and drying of the precipitates, which increases processing time and cost. Additionally, the chemicals used and the toxic gases generated during the process may have a detrimental impact on the environment. The specific reaction equation is provided below:

10Ca(NO_3_)_2_ + 6(NH_4_)_2_HPO_4_ + 2NH_4_F + NH_4_OH + 1/2O_2_→Ca_10_(PO_4_)_6_F_2_ + 14NH_4_NO_3_ + 5.5H_2_O + 7NO_2_(↑) (1)

### 2.4. Mineralization Method

Common mineralization techniques include the cryomineralization and biomineralization methods. As illustrated in Figure 3b, the cryogenic mineralization process can be broadly divided into three stages. In the first stage, a fluorine-containing simulated body fluid (F-SBF) is prepared by adding potassium fluoride to F-SBF to adjust both the F^−^ concentration and pH following the methodology outlined by Kokubo et al. [42]. Subsequently, bioactive glassy nano-powder (BGNPS), composed of SiO_2_-CaO-P_2_O_5_, is thoroughly mixed with the fluoride-enriched simulated body fluid. To activate the surface of the BGNPS, the mixture is allowed to stand for 30 min, facilitating the pre-treatment process of mineralization and leading to the formation of FGC at the particle interfaces. In the second stage, the prepared solution is poured into a mold, heated to 120 °C, and axial pressure is applied. After cooling, the sample is removed, rinsed with distilled water to eliminate excess ions, and then dried in an oven [43]. The conventional sintering densification of FGC typically occurs at temperatures ranging from 700 to 1000 °C, which are energy-intensive processes [44,45]. In contrast, the low-temperature mineralization process can achieve densification at 120 °C. This method primarily facilitates the densification of inorganic materials by promoting the formation and growth of crystalline phases at the particle interfaces through the mineralization of nanoscale particles.

Biomineralization is a widely used technique, particularly in the context of dental restoration and the prevention of pathological conditions, through biomimetic strategies [46]. Its applications can be broadly categorized into two main approaches. First, biomineralization is directly employed to biomimetically regenerate tooth enamel, as depicted in Figure 3f. Fan et al. demonstrated the formation of organized, needle-like fluorapatite crystals in the presence of amelogenin and F^−^, resulting in enamel regeneration via a remineralization process [47,48]. In a similar study, Shao et al. applied calcium phosphate-rich clusters to the surface of an artificially decayed tooth, which was subsequently immersed in a solution simulating the oral saliva environment. Within 48 h, a 2.5 μm thick crystalline restorative layer was successfully grown on the tooth surface. This layer exhibited microstructural, compositional, and mechanical properties akin to those of natural enamel, and was seamlessly integrated with the original tooth tissue [49]. Second, biomineralization is utilized to produce fluorapatite and its biomimetic regenerative nanocomposites, as outlined in Figure 3d. The process involves the addition of gelatin powder to a solution containing a fixed ratio of calcium, phosphate, and F^−^. After mixing and adjusting the pH to below 6, the solution is left to incubate, followed by the addition of CaCl_2_ and heating in a 37 °C water bath. During this process, calcium ions gradually diffuse, leading to the formation and settlement of fluorapatite powder [50]. Mg^2+^ has been shown to play a significant role in enamel formation. Li et al. synthesized a multi-layered enamel-like columnar FAp/polymer nanocomposite using a biological room-temperature mineralization technique, controlled by the presence of Mg^2+^. The results indicated that Mg^2+^ induced the compaction of the arrays and facilitated the formation of a unique Mg-rich amorphous reinforced structure [51]. The biomineralization method has proven effective in producing a microstructure resembling natural tooth enamel, offering excellent biocompatibility. Moreover, by adjusting mineralization parameters such as pH, temperature, and ionic concentrations, the composition and properties of the resulting material can be tailored to meet specific application needs. While biomineralization has advanced the field of dental filling restoration through biomimetic regeneration, it is not without limitations. The process is relatively slow, incurs high costs, and is significantly influenced by external factors, such as material quality, which can affect the consistency and efficiency of the regeneration process. The mechanical properties of different FGC synthesis methods are shown in Table 2.

## 3. Performance of FGC

In the field of dental restorations, the material properties play a critical role in determining its suitability for clinical use. For FGC, the fabrication of appropriate dental restorations and their precise matching to a patient’s specific lesion are essential steps in ensuring successful outcomes. This chapter provides a comprehensive analysis of the key properties of FGC, focusing on aspects such as biocompatibility, antimicrobial activity, resistance to degradation, aesthetic qualities, wear resistance, and mechanical properties. By thoroughly investigating these properties, we aim to enhance the effective application of FGC in restorative dentistry and facilitate their further advancement to better meet the therapeutic needs of patients.

### 3.1. Biocompatibility

Biocompatibility refers to the ability of a material to perform its intended function in medical treatment without eliciting unfavorable local or systemic reactions in the recipient. Simultaneously, it should enhance the clinically relevant properties of the treatment under specific conditions [52]. The validation of biocompatibility is typically conducted in three stages: (1) in vitro experiments, (2) in vivo animal studies, and (3) clinical trials in humans. FGC, often utilized as an oral restorative material, releases F^−^ to prevent oral diseases [53]. When employed as an implant coating, it facilitates the biological bonding of both hard and soft tissues, achieving a robust connection between the implant and surrounding tissues. This process involves a complex mechanism of ion release, controlled dissolution of the glass surface, and the formation of a precipitate layer [54,55]. Kazuz et al. utilized FGC as a root canal filling material, and in vitro cytotoxicity assays demonstrated no adverse effects on MRC-5 human fibroblast cells [56]. Similarly, Bibby et al. sputtered FGC coatings onto Ti_6_Al_4_V and Al_2_O_3_ surfaces, which, when immersed in simulated body fluid (SBF), exhibited favorable synergistic effects with bone tissue [57,58]. In another investigation, FGC was found to display a unique nanomorphology, characterized by flower-like fluorapatite crystals, which promoted the differentiation of human mesenchymal stem cells, positioning it as a promising candidate for bone regeneration ceramics [59,60]. In vivo studies in rabbits demonstrated that FGC maintained long-term implant stability and osseointegration over 26 weeks [61]. Furthermore, the biocompatibility of FGC is influenced by the fabrication process. Borkowski et al. found that sintering FGC at 800 °C optimized its porosity and F^−^-release capacity, thereby promoting osteoblast proliferation [62]. To further enhance the bone regenerative properties of FGC, researchers have optimized fabrication techniques and incorporated second-phase dopants. Denry et al. improved the sintering rate under vacuum conditions, which significantly augmented the bone regeneration capacity of FGC scaffolds [63]. Another study demonstrated that strontium-doped FGC exhibited a finer microstructure and increased solubility, leading to sustained strontium release, which in turn promoted the formation and differentiation of osteoblasts in rats [64].

Notably, in FGC, smaller fluorapatite grains are more readily internalized by cells or interact with the cell surface, facilitating adsorption, phagocytosis, or other intercellular responses. Conversely, larger fluorapatite grains may induce mechanical stress within the cell, potentially compromising cell membrane integrity or organelle function. Additionally, larger crystals may occupy more space or alter the structure of the extracellular matrix, negatively affecting cellular growth and function. Researchers have doped FGC with niobium or niobium oxide to regulate the size of fluorapatite crystals, demonstrating that human mesenchymal stem cells exhibit favorable attachment, proliferation, and differentiation on the modified glass-ceramics [65,66]. In vivo studies also indicate that fluorapatite coatings on implants offer greater stability than hydroxyapatite coatings in non-weight-bearing models [67,68]. In conclusion, FGC exhibits excellent biocompatibility, meeting the requirements for various biomaterial applications.

### 3.2. Antibacterial Activity

Dental caries remains highly prevalent, often resulting in tooth sensitivity and pain, and in severe cases, leading to pulpitis or periapical inflammation. Such conditions can impair masticatory function and overall health. Furthermore, the improper handling of dental implants post-surgery can lead to bacterial infections, which may cause complications and ultimately result in implant failure [69]. Consequently, antimicrobial properties are essential for preventing infections in both implants and the surrounding oral environment. Microcrystalline glass regulates the release rate of ions, thereby influencing local osmotic pressure and pH, which affects both the implant and the surrounding physiological conditions. As such, the antimicrobial properties of microcrystalline glass are highly dependent on its composition and solubility [70]. The mechanism of the bacteriostatic action of glass-ceramics has been classified into two primary steps: (i) the release of ions from the glass-ceramics into the surrounding medium, and (ii) the modification of the local physiological environment’s osmotic pressure and PH [71]. Specifically, FGC releases alkaline ions, particularly Ca^2+^, which disrupt the intracellular Ca^2+^ balance in bacteria. This disruption impedes cell membrane formation and induces cytotoxicity, ultimately leading to bacterial cell death [72]. Moreover, under low pH conditions, FGC releases F^−^, which inhibits bacterial growth. Fluorapatite has also been shown to enhance tooth remineralization, reduce the solubility of enamel and dentin, and thereby prevent dental caries [73]. Although the antimicrobial efficacy of FGC is somewhat limited, the breadth of bacterial inhibition and the range of susceptible bacterial species can be extended by doping the glass with specific ions that possess known antimicrobial properties, such as Zn^2+^, Sr^2+^, and Ce^3+^. For instance, the addition of ZnO_2_ to FGC has been shown to significantly inhibit the growth of Staphylococcus aureus, Bacillus cereus, Bacillus waxiformis, and Escherichia coli [74,75]. Additionally, the antimicrobial activity of FGC doped with strontium has been demonstrated against Staphylococcus aureus and Enterococcus faecalis [76,77]. Ions with antimicrobial properties, such as Ag^+^ and Cu^2+^, have also been shown to effectively disrupt Streptococcus haematobium and Porphyromonas gingivalis biofilms in the oral cavity [78,79]. The introduction of metal ions in appropriate quantities is crucial for maintaining good health; however, excessive levels can lead to adverse effects, such as allergic reactions, oral inflammation, and potential erosion, which may compromise the health of teeth and surrounding tissues. Additionally, the bacteriostatic properties of FGC are influenced by its particle and grain size, as well as its porosity and surface roughness. Generally, smaller particle and grain sizes, coupled with lower surface roughness, are associated with improved bacteriostatic performance. Conversely, increased porosity tends to enhance these properties as well.

### 3.3. Degradation Resistance

Chemical durability is a critical performance indicator for dental materials. Fluctuations in pH within the oral environment can lead to the corrosion of dental restorative materials. Such corrosion not only compromises the fracture strength of the material but also deteriorates surface roughness, ultimately resulting in abrasive wear and facilitating the onset of tooth decay [80]. Consequently, dental ceramics may experience progressive degradation due to mechanical occlusion, chemical corrosion, or a combination of both factors [81]. The corrosion resistance of FGC is influenced by pH conditions, with silica being more soluble in alkaline environments, while the alkaline components of FGC are prone to ion exchange with protons in acidic conditions [82,83]. Hsu et al. investigated the activation energy for the dissolution of FGC veneers across varying pH levels, establishing a relationship between temperature and dissolution rate, thereby enhancing its predictability for clinical applications [84].

The chemical durability of microcrystalline ceramics is closely tied to both the composition of the residual glass phase and the characteristics of the crystalline phase. To improve the chemical durability of FGC in the oral environment and maintain oral health, researchers have explored the incorporation of alternative glass-ceramic materials or substances to facilitate the nucleation and crystallization of the crystalline phases. Fathi et al. demonstrated that ZrO_2_ promoted crystallization, resulting in the formation of more stable fluorapatite phases, which in turn enhanced the chemical durability of FGC [85]. In a separate study, TiO_2_ was incorporated into apatite–mullite glass-ceramics at a concentration of 2.5 wt%. This modification resulted in a reduction in the material’s solubility by 509.8 μg/cm^2^ compared to the original composite glass-ceramics [86]. Bafghi et al. assessed the degradation resistance of fluorapatite-albite glass-ceramics, which exhibited a solubility of less than 100 μg/cm^2^, in compliance with the ISO 6872 standard for acceptable weight loss in dental ceramic materials [87]. Furthermore, Hsu et al. applied a biocompatible SiC film to FGC using plasma-enhanced chemical vapor deposition. This coating significantly reduced the solubility of the glass and minimized plaque accumulation under extreme PH conditions, thus preventing dental caries and periodontal inflammation [88]. Although the application of a SiC coating improves the chemical durability of FGC, challenges such as delamination may arise due to the inherent porosity, leading to the degradation of the material. To mitigate this issue, Fares et al. employed annealing and the plasma treatment of the coating, thereby reducing the likelihood of delamination [89]. Additional strategies to enhance degradation resistance include optimizing the synthesis ratio of CaO and MgO, controlling the crystallization process to increase the crystalline phase ratio, and employing surface modification techniques such as polishing, glazing, and bioactive coating to improve the long-term stability of FGC.

### 3.4. Aesthetic Characteristic

**Figure 4:**
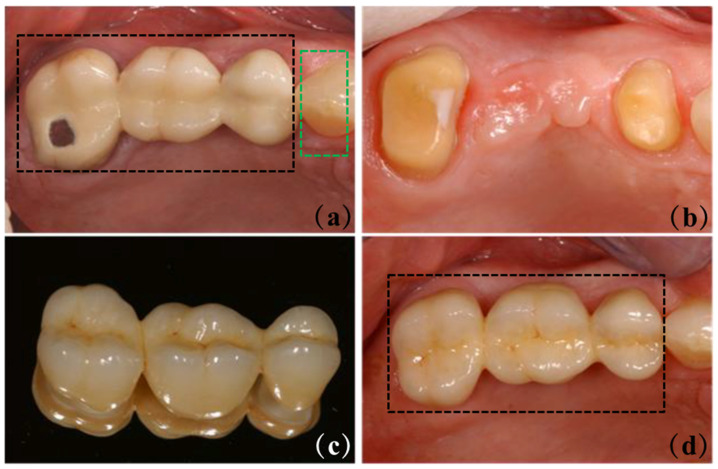
Diagram of FGC coatings established on ZrO_2_ dental bridges. (**a**) In the initial case, the black box is an uncoated ZrO_2_ bridge, and the green box is a natural tooth; (**b**) There is no dental bridge; (**c**) FGC veneer ZrO_2_ bridge; (**d**) The morphology after repair, the black box is the ZrO_2_ bridge of FGC veneer. Adapted from ref. [102].

In restorative dentistry, the optical properties of ceramic materials, including translucency and opalescence, are crucial for achieving aesthetic restorations that closely mimic the appearance of natural teeth. Semi-transparency refers to the degree to which a material allows light to pass through or scatter, representing a state between complete opacity and full transparency [90]. The milky effect, in contrast, describes an optical characteristic where a dental material appears bluish-white in reflected light and exhibits orange or brown hues in transmitted light [91]. Furthermore, Tanweer conducted erosion experiments on FGC under various solution conditions. The results indicated that the crystal structure of FGC remains stable, and the calcium, phosphorus, and fluorine elements within the material are effective in mitigating color changes induced by high temperatures and alterations in the chemical environment [92]. Additionally, rare earth ions, such as lanthanum ions, can interact with the ions present in the matrix material, thereby significantly enhancing its color stability [93]. Moreover, remineralization techniques can be employed to restore carious lesions and regain the original color stability of both the restoration and the tooth [94]. ZrO_2_, known for its high opacity and whiteness, exhibits significantly different color, brightness, and transparency compared to natural teeth. The average opalescence parameter values for tetragonal-phase ZrO_2_ range from 1.25 to 2.83, while those for human teeth fall between 4.8 and 7.4 [95,96]. To improve the aesthetic properties of ZrO_2_ restorations, a layer of FGC is often applied to the surface. As shown in Figure 4a, a damaged ceramic bridge needed to be replaced in this clinical case. Large-span bridges are typically made of high-strength ZrO_2_, a material known for its toughness properties. Compared to natural teeth (shown in the green box), ZrO_2_ bridges (prominently identified in the black box) differ from real teeth in color, brightness, translucency, and wear characteristics. However, after careful manipulation by the dental technician, based on the information contained in Table 3, an FGC coating was successfully constructed on the surface of the ZrO_2_ bridge, as shown in Figure 4d, which achieved a high degree of similarity in visual appearance to the neighboring natural teeth.

Furthermore, FGC coatings generally do not require staining due to their natural milky or creamy white hue, which complements the color of natural teeth. However, to meet specific clinical and aesthetic requirements, staining may occasionally be applied to achieve a more precise color match and uniform texture. Surface roughness and gloss retention are also critical factors in the aesthetic performance of FGCs. Increased surface roughness can lead to light scattering, negatively impacting the transparency and visual appearance of the material. This is particularly relevant in restorative dentistry, where an excessively rough surface may compromise the aesthetic harmony with natural teeth. Therefore, surface polishing and refinement are essential to reduce roughness and enhance the material’s appearance. During clinical use, FGC may experience wear, oxidation, or contamination, all of which can result in a loss of surface gloss. Maintaining surface gloss is crucial for preserving aesthetics over the long term, particularly during routine cleaning. The retention of gloss is influenced by factors such as surface roughness, the condition of the coating, and the abrasion resistance of the material. The wear resistance of FGCs can be substantially improved through careful adjustments to the chemical composition of the material and surface treatment processes, ultimately resulting in prolonged gloss retention. Moreover, the optical properties of FGCs can be further enhanced through glazing and surface polishing, contributing to an increase in gloss and surface smoothness.

While FGC is already aesthetically similar to natural teeth, ongoing research aims to improve their translucency and opalescence by examining key factors, such as thickness, internal structure, crystal content, and crystal size, to better suit various oral environments and meet personalized patient needs [97,98,99]. Studies have shown that increasing the concentrations of CaO, P_2_O_5_, and F^−^ leads to a gradual enhancement in the translucency and opalescence of FGC [100]. In another investigation, Zhang et al. incorporated Eu^3+^ into FGC, resulting in a transmittance of up to 80.5% in the visible range for a 1.92 mm thick sample [101]. The continuous evolution of FGC in aesthetic performance demonstrates significant potential in meeting the rising demand for personalization.

### 3.5. Wear Resistance

Tooth wear is a naturally occurring, inevitable process primarily resulting from occlusal contact between teeth or restorations during masticatory activities or due to abnormal oral functions [103]. The wear mechanisms between teeth or restorations can be categorized into two primary types: two-body wear and three-body wear [104]. In two-body wear, the primary mechanisms of wear in FGC and natural teeth immersed in saliva are adhesive wear and erosive wear. These result from the adhesive forces at the contact surfaces and the acidic etching effects of saliva [105]. In contrast, during actual mastication, wear often involves a combination of both two-body and three-body wear modes. In the three-body wear state, fluid lubrication is present, and the FGC and natural tooth surfaces do not directly contact each other, leading to a lower coefficient of friction and reduced wear rate compared to two-body wear [106].

When harder materials, such as ZrO_2_, are used as restorations, they may induce excessive wear on natural teeth due to their higher hardness. This can result in complications such as abnormal stress, occlusal imbalances, and tooth sensitivity [107,108]. With increasing patient demands for aesthetic outcomes, double-layer all-ceramic restorations have gained popularity. However, FGC, commonly used as veneering materials for ZrO_2_ restorations or as standalone inlays and crowns, tends to exhibit higher wear rates, which may lead to premature failure of oral restorations [109,110]. Consequently, there has been substantial research into improving the wear resistance of FGC.

**Figure 5:**
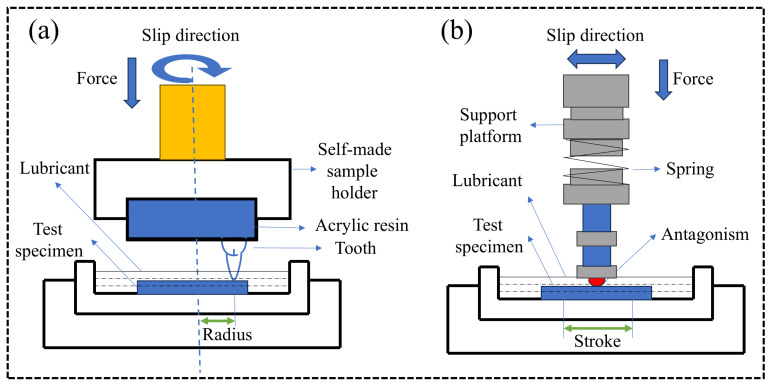
(**a**) Pin disk friction wear test; (**b**) sliding friction wear test. Adapted from ref. [106,112].

The wear resistance of a material is generally proportional to its mechanical properties. Wang et al. improved the wear resistance of FGC by optimizing the crystalline content and structural dimensions of fluorapatite crystals. This was achieved by adjusting the CaO content in the composite and refining the sintering process, which significantly reduced adhesive wear behavior, lowered the coefficient of friction, and diminished the overall wear rate [24]. Meng et al. employed an ion-exchange method to induce a residual compressive stress layer on the surface of FGC. This approach notably enhanced the mechanical strength of the material and reduced the wear rate during both the break-in and steady wear phases [111]. Additionally, Yang et al. utilized SLA 3D printing technology to modify the forming process of FGC dental restorations, exploring the primary wear mechanisms such as fatigue wear and adhesive wear [112]. Furthermore, researchers have also sought to improve the wear resistance of FGC through the development of composite materials. Tan et al. demonstrated that introducing a certain degree of porosity in FGC by incorporating calcium-mica bioactive glass powder enhanced the material’s wear resistance [113]. Other studies have focused on reinforcing FGC with hard fillers, such as Al_2_O_3_ whiskers and ZrO_2_ fibers, which have shown promising results in enhancing wear resistance [114,115]. It is important to note that results can vary depending on experimental conditions. Two commonly employed friction wear testing methods for FGC and their composites are illustrated in Figure 5, and the parameters and outcomes of these friction wear tests are summarized in Table 4.

The porosity, bonding, and intrinsic material properties of composites are critical factors influencing their wear resistance. Further research is required to quantitatively control these parameters to optimize the wear performance of these materials. While significant progress has been made in enhancing the wear resistance of FGC through improvements in heat treatment processes, forming methods, secondary strengthening techniques, and the incorporation of secondary phases, their wear resistance remains marginally lower than that of ZrO_2_, lithium disilicate (LD) glass-ceramics, and white crystalline glass-ceramics [116]. Despite these advancements, the wear resistance of FGC still presents room for improvement. Recent technological innovations offer promising avenues for enhancing the wear resistance of FGC. Microwave energy sintering, a body-centered heating technique, is one such emerging method that minimizes energy loss and facilitates uniform heating. This technique promotes the formation of a finer microstructure, which in turn can enhance the material’s wear resistance and mechanical properties when compared to conventionally crystallized ceramics. Microwave energy sintering has been extensively studied as a potential alternative to traditional firing methods in ZrO_2_ and LD glass-ceramics [117,118,119,120]. Preliminary studies indicate that microwave energy sintering may also offer significant potential in the sintering of FGC.

### 3.6. Mechanical Property

According to ISO 6872, flexural strength and fracture toughness are essential mechanical properties used to evaluate the success of dental restorations. FGC, which incorporates a fluorapatite crystalline phase within an amorphous matrix, exhibits superior mechanical properties compared to uncrystallized base glass. Through the continuous optimization of the sintering process—including adjustments to heating rate, crystallization temperature, and holding time—along with modifications to the composite composition, researchers have been able to promote the growth of fluorapatite grains, increase the number of crystalline phases, and reduce porosity. These improvements enhance the mechanical properties of pure FGC. However, due to the inherent limitations of the material, the strength of pure FGC, typically molded using pressing methods or 3D printing technology, ranges from 40 to 160 MPa, and the fracture toughness spans from 0.85 to 1.7 Mpa·m^1/2^ [24,25,121,122]. Consequently, the strength and fracture toughness of pure FGC meet the performance requirements for various dental applications, including veneers, inlays, onlays, double-layered all-ceramics, and filling materials, as summarized in Table 5.

The modulus of elasticity and hardness are also critical mechanical indicators in restorative dentistry, as they influence the mechanical compatibility of restorations with natural teeth and bone. A significant mismatch in the modulus of elasticity between an implant and the surrounding bone can lead to undesirable bone resorption. As shown in Table 4, FGC has a modulus of elasticity ranging from 60 to 97 GPa, making them suitable for use as coatings on implants and other dental inserts, such as ZrO_2_, Ti, and their alloys (with moduli of elasticity between 120 and 220 GPa). This application helps to reduce the modulus of elasticity mismatch between the implant and the bone, thus mitigating the risk of bone resorption.

Additionally, as demonstrated in Table 6, the mechanical properties of FGC are somewhat less favorable compared to other dental materials, such as ZrO_2_, Al_2_O_3_, and LD glass-ceramic. However, in terms of aesthetics, FGC offers notable advantages, particularly in its exceptional translucency and color adjustability, which significantly outperform ZrO_2_, Al_2_O_3_, and LD glass-ceramic. The study demonstrates that FGC exhibits superior thermal stability, a low coefficient of thermal expansion, and excellent resistance to thermal fatigue during thermal cycling experiments, effectively preventing the initiation of cracks and fractures [123]. In contrast, brittle ceramics, owing to their high coefficient of thermal expansion, are more susceptible to cracking and rupture under thermal stress, exhibiting relatively poor thermal fatigue resistance. Consequently, FGC displays distinct advantages in environments subjected to thermal cycling [124].

## 4. The Forming Process of FGC Dental Restorations

**Figure 6:**
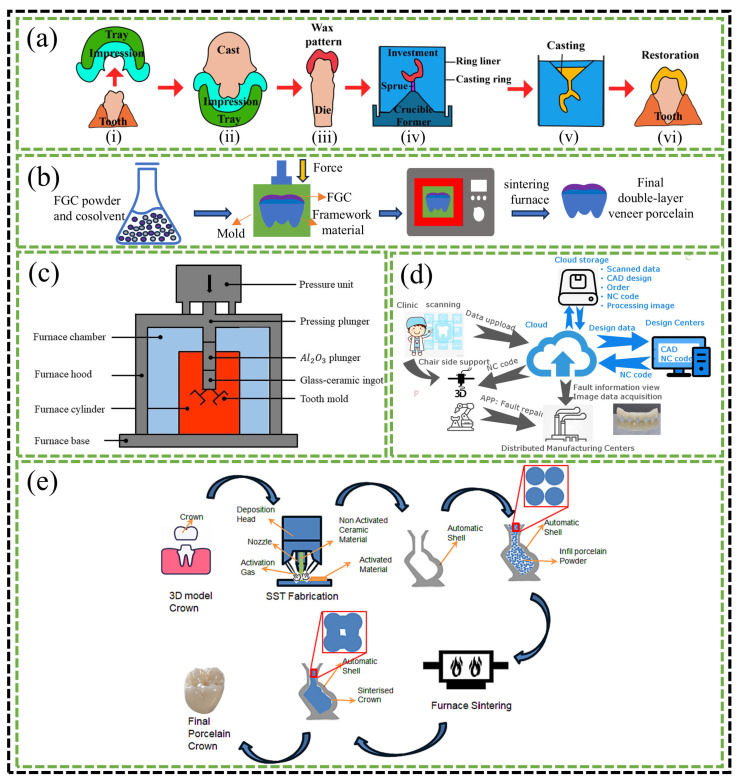
Forming process for FGC dental restorations. (**a**) Lost wax technique; (**b**) pressureless sintering; (**c**) hot press sintering; (**d**) CAD/CAM; (**e**) shell sintering. Adapted from ref. [151,152,153]. (Note: CAD/CAM technology is not usually used alone for the production of FGC dental restorations, but more as an auxiliary process for the fine grinding of the restorations or machining composite double-layered ceramic framework materials such as ZrO_2_ and LD glass-ceramic).

In restorative dentistry, the forming process plays a critical role in determining both the performance and the manufacturing costs of dental restorations. The selection of an appropriate forming technique, tailored to the specific characteristics of the different types of restorations, not only ensures optimal performance but also enhances material efficiency while reducing time and cost. As illustrated in Figure 6, this chapter provides a comprehensive overview of the various forming processes used for FGC dental restorations. These processes include lost wax, pressureless sintering, hot pressing, CAD/CAM milling, additive manufacturing (AM), and shell sintering. Through a detailed analysis of the advantages and limitations of each method, this chapter seeks to refine the existing processes and explore novel forming techniques, with the ultimate goal of advancing industrial technologies and fostering a closer integration of research, industry, and market demands.

### 4.1. Lost Wax

The lost wax technique is widely employed in the fabrication of complex dental restorations, including veneers, inlays, and crowns. The process begins with obtaining an accurate impression of the tooth (Figure 6(ai)), which serves as the foundation for creating the wax model (Figure 6(aii)). A wax mold of the target restoration is then formed on the casting model using wax materials (Figure 6(aiii)). Subsequently, the wax mold is coated with a bonding agent and a heat-resistant material (Figure 6(aiv)) [151]. The wax mold is then subjected to burnout at approximately 900 °C. During a subsequent high-temperature heat treatment (1300–1500 °C), the FGC is introduced into the mold, displacing the wax mold, which has been replaced by a binder and refractory material (Figure 6(av,avi)). After cooling, the restoration is polished and cleaned to yield the desired glass-ceramic restoration. To ensure the restoration closely matches the natural tooth appearance, it is often colored at the final stage. Additionally, the lost wax technique is also utilized in the fabrication of veneer porcelain via a pressing method, where a wax mold is placed over the framework and injected with FGC, followed by the removal of the wax mold [154,155].

The lost wax technique is capable of producing structurally complex and clinically suitable dental restorations at relatively low manufacturing costs, with the added benefit of ease of handling. Generally, the method is well suited for fabricating restorations made of low-melting-point materials such as FGC. However, there are some inherent limitations. Since FGC requires crystallization via a surface mechanism, the forming process must undergo specific heat treatments to facilitate crystal formation. The presence of wax molds can result in uneven heat distribution, which may adversely affect the microstructure and properties of the final restoration [8]. Furthermore, the dimensional accuracy of the fabricated restorations can be achieved within 20 μm, provided that the prescribed guidelines for each process step are strictly adhered to and improper handling is avoided [156].

### 4.2. Pressureless Sintering

To adjust the final color and appearance of restorative materials such as ZrO_2_, metal, or glass-ceramics, a 0.3–2 mm thick layer of FGC is often applied to the surface. This process is typically carried out through a pressureless sintering method to produce the veneer. As illustrated in Figure 6b, the pressureless sintering process begins with the homogeneous mixing of fluorapatite powder with a glass-phase co-solvent, according to a specified formulation. The resulting powder mixture is then placed into a mold for initial pressing using a cold isostatic press. Following this, the mold is placed in a sintering furnace, where it undergoes sintering at a predetermined temperature. After sintering, the mold is allowed to cool within the furnace, and subsequent after-treatment operations, including sanding and polishing, are performed to achieve the desired finish [151,157].

The veneered porcelain produced via pressureless sintering exhibits excellent mechanical properties and can be easily processed for secondary operations. However, the pressureless sintering process requires precise control of several parameters, including the heating rate, the self-weight of the material, and the sintering atmosphere to prevent the development of thermal stresses and cracks. Additionally, proper control is essential to enhance the densification of the material. To ensure that the final veneered porcelain meets the rigorous standards for clinical use, fine finishing operations such as cutting, grinding, and polishing are necessary. These steps are critical for ensuring an optimal fit and aesthetic appearance of the veneer. Moreover, coating and glossy surface treatments are often applied to improve both the aesthetic qualities and wear resistance of the veneered porcelain. These additional processes, while necessary, increase the complexity of the overall procedure.

### 4.3. Hot Pressing

The hot pressing process remains the predominant technology for the fabrication of glass-ceramic dental restorations, such as crowns, inlays, and veneers, in the current market. This process utilizes pre-prepared glass-ceramic ingots to form the desired restorations. The initial steps closely resemble those of the lost wax method, which involves the creation of a mold for the restoration [158,159]. Subsequently, the prefabricated glass-ceramic block or powder is placed into a specialized furnace, as shown in Figure 6c, along with the mold. At elevated temperatures, the glass-ceramic ingot or powder transforms into a viscous fluid state, which is then injected into the mold by applying pressure through a plunger. Once the material cools, post-treatment procedures, such as sanding with sandpaper, are employed to remove surface impurities from the restoration [151]. Gorman et al. [160,161] have established the optimal temperature and heat treatment duration for the hot pressing of FGC, ensuring the material attains the desirable mechanical properties. Furthermore, other types of glass-ceramics, including LD, albite, feldspar, and mica-based compositions, have also been successfully processed into various dental restorations using the hot pressing technique [162,163,164,165,166,167,168].

In comparison to pressureless sintering, the hot pressing process offers the advantage of minimal shrinkage during crystallization, which significantly reduces sintering time. As a result, dental glass-ceramics produced by hot pressing exhibit fewer structural defects, superior mechanical properties, and enhanced edge fit [165,169].

### 4.4. CAD/CAM

The operational process of CAD/CAM milling technology in clinical applications is illustrated in Figure 6d. Initially, the tomographic imaging of the tooth and surrounding tissues to be restored is performed using a scanner or CT. The acquired image data are subsequently transmitted to a computer. A virtual reconstruction of the missing part is carried out using specialized software, and the resulting model data are then sent to the CAD/CAM machine. The milling tool proceeds to carve the target model from a prefabricated glass-ceramic material, following a predetermined path [152].

Bilayer ceramic restorations typically consist of a core material and a veneering ceramic, with the CAD/CAM milling process serving as the primary method for manufacturing such restorations. Among the various core materials available, ZrO_2_ is particularly favored for its outstanding mechanical properties. FGC, on the other hand, is often chosen as veneering ceramics due to their aesthetic and functional advantages. There are two main approaches for fabricating double-layered veneer ceramics. The first involves separately milling the ZrO_2_ core and fluorapatite veneer using CAD/CAM technology, followed by combining the FGC with the core material through layering, pressing, or other techniques [99]. The second method involves pressing and sintering the FGC onto ZrO_2_ to form a solid unit, which is then fine-tuned and shaped using the CAD/CAM milling process. The former method is known for its high precision and excellent fit, whereas the latter is more suitable for large-scale production.

Despite the widespread use of CAD/CAM milling in restorative dentistry, certain limitations persist. As a material removal process, CAD/CAM milling generates significant material waste. Additionally, the complexity of the structure being machined, along with the variability in dimensions and material properties, can impose constraints on the milling technique [170]. During the milling process, ceramic surfaces are prone to stress concentration, which can lead to crack propagation and chip formation, thereby increasing surface roughness [171,172,173,174] and causing both surface and subsurface damage [175,176]. FGC is particularly susceptible to such damage accumulation due to their relatively lower mechanical properties. Furthermore, veneered ceramics often require clinical adjustments, typically achieved through diamond rotary instruments. However, these abrasives can similarly introduce surface defects [177,178,179,180,181,182]. Consequently, the strength of milled or ground restorations may differ significantly from the theoretical strength of the material, owing to surface and subsurface defects, stress concentration points, and residual stress distribution. This may ultimately result in clinical restoration failure [163,183,184,185,186,187]. Therefore, the development of alternative forming techniques is urgently needed to address these challenges.

### 4.5. Additive Manufacturing (AM)

Traditional fabrication methods such as lost wax casting, pressing, and CAD-CAM milling have been employed for many years in the preparation of FGC restorations. However, these techniques exhibit limitations in terms of preparation flexibility, material utilization, machining efficiency, and forming accuracy, and they are often inadequate for producing restorations with complex geometries [188]. To address these shortcomings of conventional processing methods, AM technology has emerged as a promising alternative. The AM process involves the design of a target model using computer-aided design software. The model is then sliced into multiple two-dimensional cross-sections by slicing software, followed by layer-by-layer material deposition based on a predefined path. This results in the creation of a solid three-dimensional structure that conforms to the target model [189].

**Figure 7:**
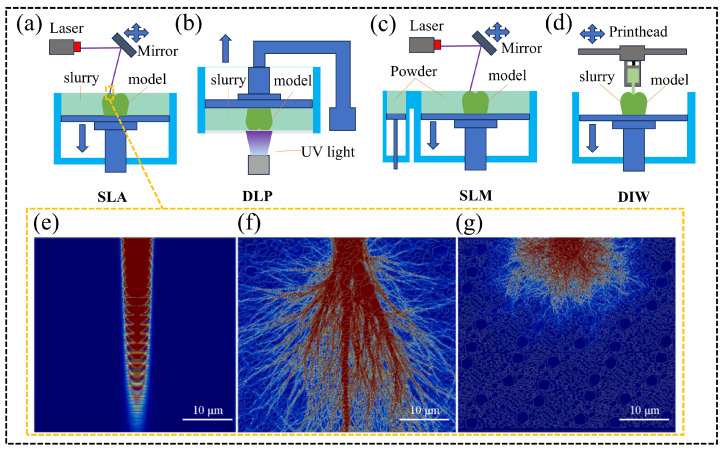
Schematic of additive manufacturing technologies. (**a**) SLA; (**b**) DLP; (**c**) SLM; (**d**) DIW. Light scattering schematic. (**e**) Unfilled resin; (**f**) slurry containing FGC particles; (**g**) slurry mixing FGC and ZrO_2_ particles. Adapted from ref. [127].

AM has shown significant potential for producing highly customized dental restorations, offering advantages such as color gradients, high precision, and translucency. Commonly employed additive manufacturing techniques for dental ceramics include stereolithography (SLA; Figure 7a), digital light processing (DLP; Figure 7b), selective laser sintering (SLM; Figure 7c), and direct ink writing (DIW; Figure 7d) [189]. Yang et al. successfully fabricated FGC dental restorations using SLA. Under optimized printing parameters, the mechanical properties of the resulting restorations were found to surpass those produced by traditional dry-pressing methods [109]. Similarly, Shen utilized SLA printing to produce FGC and ZrO_2_ composite restorations, which featured an intra-layer gradient structure, achieving a natural color transition [190]. FGC primarily comprises SiO_2_, Al_2_O_3_, Na_2_O, and CaO, and these materials exhibit low absorbance and refractive indices that differ from those of resins. During SLA printing, this mismatch can lead to light scattering phenomena (Figure 7f), where the actual curing area exceeds the programmed range, resulting in a loss of forming accuracy [191]. To mitigate this issue, Wang et al. introduced low-transmittance ZrO_2_ particles into FGC, significantly reducing light scattering (Figure 7g) and reducing dimensional errors in the forming of dental restorations by approximately 80% [192]. Additionally, Elraggal et al. demonstrated the use of the SLM technique to fuse fluorapatite veneer porcelain (FVP) onto ZrO_2_ frameworks, achieving superior bonding strength compared to the traditional sandblasting methods applied to silica-covered Al_2_O_3_ [193]. DIW is a material extrusion technique that holds considerable promise for the forming of FGC. This method utilizes gel-based inks that can be degreased at temperatures below the glass transition point, resulting in highly dense structures [194]. Although DLP and SLA are more accurate than SLM and DIW, these methods face significant challenges in producing multi-material gradient structures, especially intra-layer gradients. The fabrication of such structures often requires multiple slurry tanks or material switching, increasing both equipment costs and printing times. In contrast, the extrusion of different inks simultaneously or sequentially in DIW offers greater flexibility for the creation of complex multi-material structures [7].

CAD/CAM milling technology offers significant advantages in terms of precision, particularly in the processing of ceramic restorations, which enhances their strength and stability. However, the inherent complexity of the milling process, coupled with material waste and the requirement for high-precision equipment, often results in elevated production costs. In contrast, AM provides greater design flexibility and the ability to fabricate more intricate geometries, particularly in multi-layered structures, fine-tuned designs, and customized restorations. While AM generally lacks the same level of strength and precision as milling, it presents notable cost-control benefits, particularly in personalized and low-volume production scenarios. Although the process is slower, AM is particularly well suited for complex designs and restorations with specialized needs. In certain restoration manufacturing contexts, hybrid processes that combine CAD/CAM milling and AM technologies can harness the strengths of both techniques. For instance, additive manufacturing can be employed to fabricate the primary structure of a complex restoration, which can then undergo precision machining through CAD/CAM milling. In conclusion, despite the differences in operational processes, application areas, and technical characteristics between CAD/CAM milling and AM, both technologies are playing an increasingly significant role in the field of dental restorations. With ongoing technological advancements and innovations, it is anticipated that a more seamless integration of these methods will emerge, offering patients more precise, efficient, and personalized restorative solutions.

### 4.6. Shell Sintering

In the field of dental restoration manufacturing, a variety of advanced processes have been employed to fabricate FGC restorations in addition to the conventional forming techniques previously discussed. Recently, Cocco et al. introduced a shell sintering method as a response to the United Nations’ sustainability and quality objectives. This technique utilizes FGC as the primary material for the production of dental crowns. The specific details of the manufacturing process are illustrated in Figure 6e. The findings from their study indicate that this shell sintering approach is a viable and effective method for producing high-quality FGC dental restorations [153].

## 5. Application of FGC in Dentistry

### 5.1. FGC as a Veneer Material

**Figure 8:**
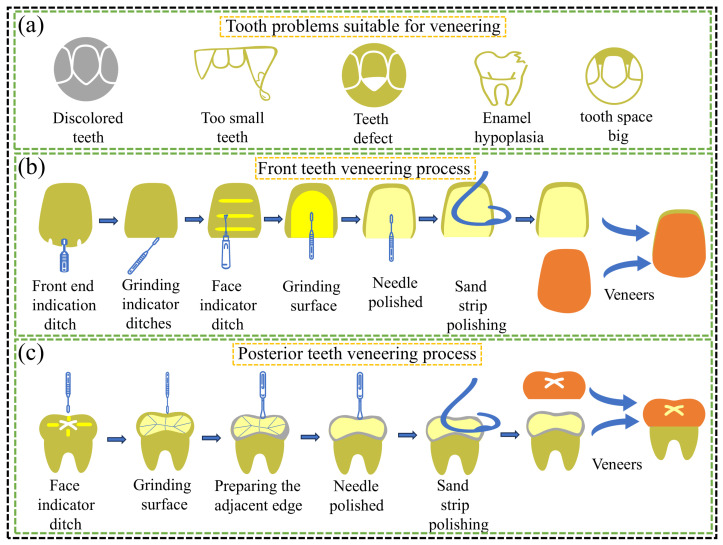
(**a**) Unhealthy teeth suitable for veneers; (**b**) process flow for anterior veneers; (**c**) process flow for posterior veneers.

FGC veneers are extensively used to address a range of aesthetic and functional issues, including the discoloration of anterior and posterior teeth, overbite, enamel hypoplasia, and excessive gaps, as shown in Figure 8a. The primary processes involved in the veneering procedure are illustrated in Figure 8b,c. In the early 21st century, FGC was predominantly applied to metal-based restorations, such as overlay inlays. For instance, Holand et al. employed FGC veneers on metal anterior teeth, achieving a visual match with adjacent natural teeth [23]. With the increased adoption of ZrO_2_ in dental restorations, due to its superior properties compared to traditional metal-based materials, FGC has gained widespread use as veneering materials for ZrO_2_ restorations and as finishing porcelains for bridge constructions [195,196,197]. Notably, Spies et al. demonstrated that FGC veneers performed effectively as zirconia veneers, meeting the patient’s needs over 30 months in the oral cavity [198]. Additionally, Ritzberger applied FGC veneers to three-unit ZrO_2_ bridges, achieving restorations that closely resembled the appearance of neighboring natural teeth, thus fulfilling the patient’s aesthetic requirements [99]. Moreover, researchers have explored the use of FGC as veneering materials with enhanced mechanical properties. However, it has been noted that applying FGC as veneers on LD glass-ceramic posterior crowns may lead to increased wear of the artificial crowns in comparison to the natural teeth, particularly on occlusal surfaces, suggesting that such applications should be avoided on these surfaces [199]. Despite these challenges, considering their tribological compatibility, aesthetic qualities, and biocompatibility, the clinical application of FGC as veneering porcelain remains widespread. Nevertheless, certain issues continue to require further investigation and resolution.

### 5.2. Veneer Technology and Enhancement of Veneer Porcelain Performance

The clinical performance of FGC veneers is often compromised by several factors, including the mismatch in thermal expansion coefficients between the framework material and the veneer porcelain, differences in modulus of elasticity, the presence of pore regions, inherent mechanical defects, the inadequate infiltration of the veneer material into the framework, and insufficient support from the underlying framework [200,201,202,203]. These issues frequently result in the clinical detachment of the veneer ceramics from the substrate and the fragmentation of the veneer, both of which can ultimately lead to restoration failure [201]. Zhang et al. compared the tribological compatibility of various veneer glass-ceramics, including LD, leucite-enhanced (LEU), feldspathic (FEL), and FGC, and found that while FGC exhibited superior performance compared to LD and LEU, they were more prone to fracture failure [204]. Therefore, it is critical to enhance the bond strength between the veneer ceramics and the substrate to reduce the risk of veneer fracture and improve the overall longevity of restorations.

#### 5.2.1. Effect of the Veneering Method on the Bonding Properties of FGC and ZrO_2_

**Figure 9:**
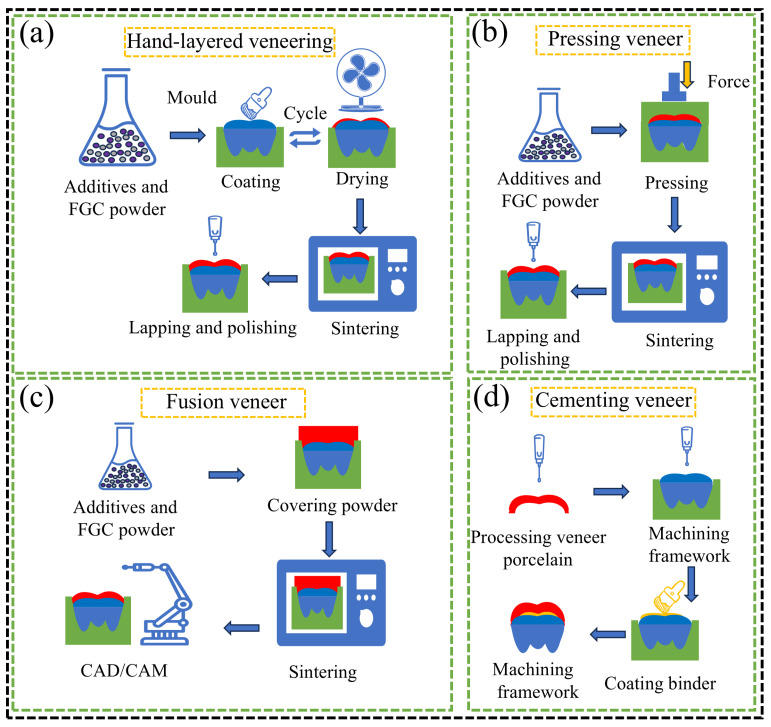
Schematic diagram of the veneering technique. (**a**) Hand coating; (**b**) hot pressing; (**c**) CAD/CAM; (**d**) cementing technology.

It has been observed that the method used to prepare veneered ceramic restorations significantly influences their damage load. Currently, the primary techniques for bonding veneered ceramics to substrates include hand layering, pressing, melt-fit CAD/CAM, and cementation (Figure 9) [205]. Since both the veneer and the core are milled from industrially prefabricated blocks, this approach reduces processing defects and sintering-related issues. In this context, the bonding of the veneer porcelain to the core typically requires only a single sintering cycle, whereas hand-layering techniques necessitate multiple sintering steps, which result in greater shrinkage. Consequently, the CAD/CAM fusion method generally exhibits higher bond strength compared to hand layering [206,207,208]. While the bond strengths of hand layering and pressing techniques are similar [205], it has also been demonstrated that pressing FGC onto ZrO_2_ substrates yields a higher damage load than the hand layering method [209]. On the other hand, the bond strength achieved with the cemented veneer technique is the lowest. This is primarily due to the high crystallinity of ZrO_2_, which resists acid etching, making it challenging to establish a strong bond between the veneered porcelain and the ZrO_2_ using resin cement. As a result, the cemented method produces lower interfacial adhesion when compared to the hand-layered technique [210]. Overall, the CAD/CAM fusion technique for veneering offers the most promising approach for achieving higher bond strengths in ceramic restorations.

#### 5.2.2. Effect of Surface Treatment on the Bonding Properties of FGC and ZrO_2_

Given the importance of compatibility between the core and veneer materials, researchers have proposed various methods for treating the ZrO_2_ surface, either through mechanical or chemical modification, to enhance adhesion. Three primary surface treatment strategies have been identified for fully sintered Y-TZP blocks: (1) connectors, which typically consist of adhesives or other fixation materials used to bond the veneered porcelain to the restorative surface [211]; (2) liners, such as 46SP6 and AP40 glass, which are used as intermediate layers to improve the fit between the veneered ceramic and the tooth structure [195]; and (3) washout layers, which generally refer to protective layers, such as silica, applied to the surface of the veneered ceramic [212]. Studies have shown that after the FGC veneering of ZrO_2_, the use of a connector treatment is more effective in enhancing the bond between the core and the veneer [211]. Conversely, the use of a liner as an intermediate layer between the ZrO_2_ and the veneered ceramic may either weaken adhesion strength or have minimal impact on the bond strength [213,214].

Additional surface modification techniques, such as Al_2_O_3_ sandblasting, surface polishing, laser etching, or acid etching, aim to clean the ZrO_2_ surface, increase surface roughness, and improve the wettability of the contact surfaces, thereby enhancing adhesion. For instance, the air particle abrasion of FGC and ZrO_2_ surfaces has been found to increase surface irregularities, providing more extensive micromechanical interlocking areas for the veneer material, thus improving bond strength [193,215,216]. However, Fischer et al. concluded that sandblasting may not always enhance the bond strength of veneered ceramics. In some cases, it can even reduce fracture toughness, as sandblasting may induce surface defects that create stress concentrations and compromise the material’s integrity [201,217].

#### 5.2.3. Measures to Reduce the Fragmentation of FGC

ZrO_2_-based restorations exhibit a 21–32% incidence of veneer porcelain fracture over a 5–10-year period, whereas metal-based ceramic restorations have a significantly lower probability of veneer porcelain failure, reported at only 2.9% [218,219,220]. To mitigate the likelihood of veneer porcelain breakage, Meng et al. successfully developed an ion-exchange layer with minimal stress relaxation and considerable depth by subjecting fluorapatite veneer ceramics to ion-exchange treatment. This process enhanced the toughness of the porcelain and markedly improved its reliability [108]. In addition, Javadpour et al. synthesized fluorapatite–mullite composite glass-ceramics, which significantly enhanced the mechanical properties of FVP [221].

Currently, the processing methods for FVP and the surface treatments designed to improve the bond strength of veneer porcelain typically rely on the use of FGC as a secondary-phase material. This material is bonded to the underlying framework via secondary processing, a practice that can limit bond strength and complicate the manufacturing process. In clinical applications, such as in the restoration of natural teeth with veneers and fillings, there remains a critical need for new techniques to improve the bond strength of FVP to natural tooth structures. One promising approach may lie in SLM using 3D printing technology. In vitro studies suggest that 3D printing holds significant potential, particularly for the fabrication of core materials with complex structural surfaces. This technology can enhance the bond strength between veneer ceramics and core materials, potentially eliminating the need for secondary surface treatments. Furthermore, integrated multi-material forming technologies based on 3D printing show great promise in dental applications. Biomimetic and bio-inspired composites, such as pearl-like materials, have garnered considerable attention for their ability to replicate the microstructure of natural teeth [222]. Looking ahead, integrated multi-material forming techniques, coupled with 3D printing, are expected to facilitate the AM of FGC biomimetic composite veneer porcelain. This advancement could enable the precise replication of the mechanical and aesthetic gradients inherent in natural teeth by strategically arranging FGC and matrix materials in a graded manner. Such an approach would address the current challenges of veneer porcelain delamination and fragmentation, thereby offering a more reliable and durable solution for dental restorations.

## 6. Composite Material

Composites are materials formed by combining two or more distinct constituents, resulting in unique properties that are not present in any single constituent material. These enhanced properties are tailored to meet the specific requirements of a given application. In the field of dentistry, ceramic composites are typically created by blending ceramics with polymers or other ceramic materials [223]. The primary categories of FGC composites include uniformly doped composites and composites with graded functionality.

### 6.1. Uniformly Doped Composites for FGC

**Figure 10:**
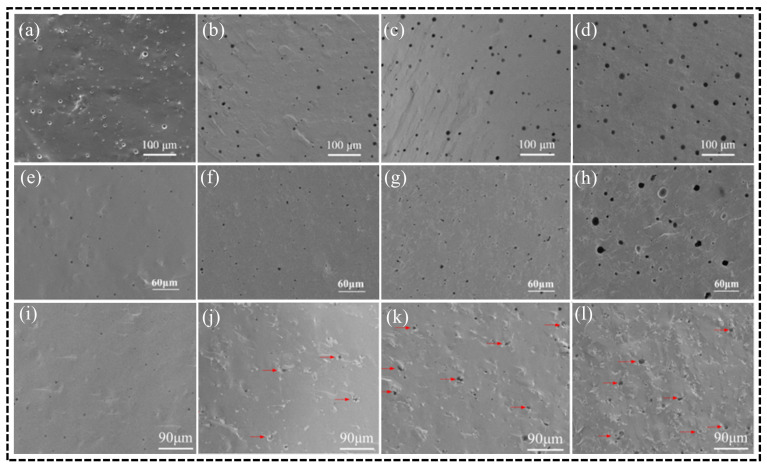
Microstructural characterization of the fracture surface of the second phase reinforced FGC. (**a**,**e**,**i**) The fracture surfaces of pure FGC; (**b**–**d**) ZrO_2_ particles; (**f**–**h**) Al_2_O_3_ whiskers; (**j**–**l**) ZrO_2_ fibers. The red arrow marks part of the gap position. Adapted from ref. [111,115,127].

Homogeneously doped composites are widely utilized to enhance the overall performance of materials by ensuring a uniform distribution of the reinforcing phase within the matrix. One of the primary limitations of FGC as repair materials is their inherent lack of strength and toughness. Research has demonstrated that the incorporation of doping particles, whiskers, and fibers into the ceramic matrix can significantly improve both the strength and toughness of the material [224,225]. In this context, researchers have successfully doped ZrO_2_ particles and Al_2_O_3_ whiskers into FGC powder, resulting in a notable increase in mechanical properties, with flexural strength and fracture toughness reaching 240.8 MPa and 3.49 MPa·m^1^/^2^, respectively. The addition of these secondary phases promotes strengthening and toughening mechanisms such as load transfer, pull-out behavior, crack pinning, as well as crack flexing, branching, and bridging, all of which contribute to a marked improvement in the flexural strength and fracture toughness of FGC [111,127,226]. These ZrO_2_ particle-reinforced composites have achieved mechanical performance on par with that of LD glass-ceramics, enabling their application in a broader range of dental restorations, including veneers, high inlays, onlays, and full crowns. This represents a significant advancement over pure FGC, which is not suitable for full crowns in clinical practice. However, as illustrated in Figure 10, pore defects remain a critical factor limiting further improvements in the mechanical properties of these composites. Thus, minimizing defects such as porosity is crucial for enhancing the performance of these materials. As presented in Table 4, while the mechanical properties of pure FGC and its composites surpass those of albite-based and mica-based glass-ceramics, they still fall short when compared to ZrO_2_, Al_2_O_3_, and LD glass-ceramics. Therefore, further enhancement of the mechanical properties of FGC, along with their expanded application in dental restorations, remains a significant area for future research. Si_3_N_4_ and SiC have already been clinically validated for use in dental implants and coatings [227,228]. Their whiskers and fibers, known for their high strength, are commonly employed to reinforce ceramics through both additive and in situ growth methods. These materials are expected to play an important role in the future reinforcement of FGC. Additionally, Dong et al. introduced the dislocation strengthening mechanism from metallic materials into ceramics, overcoming the inherent difficulty of dislocation formation in ceramics. This breakthrough represents a significant advancement in materials science and is expected to have widespread applications in ceramic materials in the future [229].

In addition to the challenge of secondary caries, one of the major concerns with dental filling materials is the strength and distribution of these materials within the resin matrix. FGC, known for its ability to release F^−^, offers a potential solution. The incorporation of porous particles in these materials increases the surface area for interaction with the resin, thereby addressing the issue of secondary caries while simultaneously improving the bond strength of the composite [230]. Furthermore, in a study by Mollazadeh et al., fluorapatite–mullite glass-ceramics were synthesized by varying the raw materials, and the resulting mullite crystals demonstrated superior mechanical properties compared to fluorapatite crystals, significantly enhancing the strength of the composites [222]. In another investigation, Ghosh et al. added 1.7 mol% of nano-ZrO_2_ to the composition of FGC, revealing that ZrO_2_ promoted cellular activity, thereby contributing to improved material performance [231]. Additionally, Höland et al. introduced a white crystal phase, KAlSi_2_O_6_, into FGC to reduce the thermal expansion coefficient mismatch between the veneering ceramics and the underlying framework, thereby enhancing the compatibility between fluorapatite and ZrO_2_ [232]. To further improve the initial bond strength of glass ionomer cement and enhance their F^−^-releasing capacity to mitigate enamel demineralization in orthodontic applications, Lin et al. incorporated fluorapatite into resin-modified glass ionomer cement, which were subsequently used for bonding composite blocks and orthodontic brackets to human enamel [233]. In summary, the combination of FGC with other materials offers a promising approach to overcoming the inherent limitations of these materials.

### 6.2. Functional Gradient Composites for FGC

In order to address the specific requirements of teeth for masticatory functions, researchers are focused on developing dental restorations that exhibit optimal biocompatibility, aesthetics, antimicrobial properties, and mechanical and tribological characteristics. These restorations are designed based on the structure, composition, and biological functions of natural teeth to meet the criteria for clinical application. Gradient composites have been engineered to accommodate more complex applications by proportionally distributing reinforcing phases across different regions, thereby creating a gradient of properties. Recognizing that the translucency of natural teeth decreases from the outer to the inner layers, Shen and colleagues employed AM technology to create an FGC functional gradient materials denture incorporating ZrO_2_, thereby replicating the structural characteristics of natural teeth. By varying the FGC content from the outer to the inner layers, they effectively reproduced the natural translucency gradient found in teeth [189]. The combination of functionally graded structure and AM technology provides a new idea for the innovative development of FGC dental restoration materials.

## 7. FGC as Dental Coating

### 7.1. Application of FGC as Dental Coating

Functional dental ceramic coatings are specifically engineered to address the inherent limitations of conventional dental restorations. These coatings serve as multifunctional materials, applied to a wide array of dental substrates, including crowns, bridges, implants, inlays, and veneers. The primary objective of these coatings is to improve the biocompatibility, antimicrobial properties, and degradation resistance of the restorations. To further optimize their performance, natural polymers, such as chitosan, are frequently incorporated, enhancing the overall efficacy and durability of the coatings. For instance, researchers have successfully prepared FGC coatings on Ti_6_Al_4_V substrates through dip-coating and heat treatment techniques. These coatings demonstrated excellent bioactivity and exhibited a defect- and crack-free interface, making them suitable for applications in Ti-based implants for human use [234,235]. To prevent the detachment of ZrO_2_ restorations, Zhang et al. employed FGC to infiltrate and coat ZrO_2_ surfaces, significantly enhancing the bond strength between ZrO_2_ and natural teeth. Their study reported an approximately threefold increase in bond strength compared to the original bond, as well as improvements in the durability and flexural strength of the adhesive bond [236]. To further strengthen the adhesion of FGC to substrates, fluorapatite–mullite composite glass-ceramics were prepared and subjected to heat treatment at elevated temperatures, which facilitated the deeper infiltration of the coating into the substrate material [57,237]. Additionally, Dunne et al. employed the CoBlast process to deposit fluorapatite onto Ti dental threaded nails. This not only promoted osseointegration but also addressed the issue of implant loosening, which is often associated with the susceptibility of hydroxyapatite coatings to delamination and dissolution. In another study, Bai et al. prepared a nano-fluorapatite coating on an osseointegrated Ti substrate using a suspension plasma process. This coating was chemically stable, and antimicrobial, and provided enhanced protection to the underlying Ti substrate [238]. To further improve the antimicrobial properties of FGC coatings, Fathi et al. incorporated ZnO into the formulation. Their experiments demonstrated that the modified glass-ceramic layer significantly inhibited the corrosion of Ti in SBF [74]. Other researchers have employed dip-coating and heat treatment processes to apply FGC onto Al_2_O_3_ and ZrO_2_ surfaces. The resulting coatings exhibited strong bonding, chemical durability, and mechanical resistance, with promising osseointegration potential and low inflammation risk for both dental and orthopedic implants [58,236,239]. Furthermore, Fayad et al. utilized electrophoretic deposition to coat Ti and micro-arc oxidized AZ91 magnesium alloy with FGC, enhancing the bioactivity and corrosion resistance of the substrate surfaces [240,241]. Additionally, direct resin composites are widely used by clinicians to repair defects and delamination sites in the oral cavity [242]. However, the combination of resin and ZrO_2_ remains a clinical challenge [243]. To address this issue, Alaaeldin et al. developed an FGC coating on ZrO_2_ surfaces using air particle abrasion and laser irradiation techniques. This approach significantly reduced damage to the restorations during the restorative process and improved the bond strength and fatigue resistance of composite resins to ZrO_2_ [244].

In addition, surface treatments and coatings play a crucial role in the aesthetic longevity of FGC restorations. These treatments not only improve the mechanical properties of the material but also contribute to its long-term appearance. Over time, dental restorations are exposed to a variety of environmental factors such as chewing forces, thermal changes, and staining agents, all of which can impair their visual appeal. Surface treatments, including polishing, enameling, and the application of protective coatings, help mitigate these effects by creating smoother surfaces, reducing wear, and preventing discoloration. In addition, coatings can provide a barrier against harmful external elements, thereby maintaining the color and luster of the restoration for a longer period. Thus, surface modifications and coatings significantly help maintain the aesthetic quality and longevity of FGC restorations, ensuring their continued success in clinical applications.

### 7.2. Preparation Technology of FGC Coating

**Figure 11:**
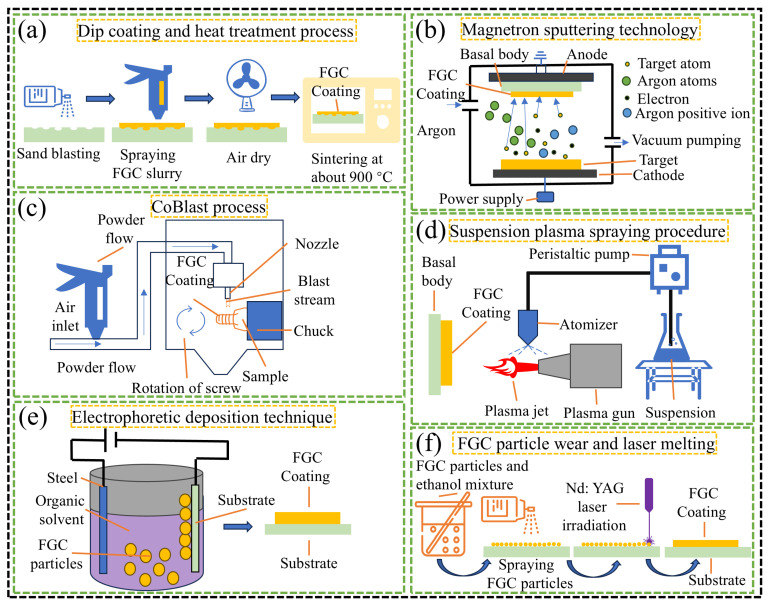
Schematic diagram of FGC coating preparation techniques. (**a**) Dip coating and heat treatment process; (**b**) magnetron sputtering technique; (**c**) CoBlast process; (**d**) suspension plasma process; (**e**) electrophoretic deposition technology; (**f**) FGC particle abrasion and laser fusion.

Various techniques are available for the preparation of FGC coatings, with the choice of method largely dependent on the specific requirements of the application. The selection of an appropriate preparation technique is guided by factors such as the intended application scenario and the desired material properties. A comprehensive exploration and detailed understanding of these coating preparation methods enable researchers to optimize the fabrication of FGC coatings tailored to meet specific performance criteria. As illustrated in Figure 11, several commonly employed techniques for preparing FGC coatings in the dental field are presented and discussed.

#### 7.2.1. Dip Coating and Heat Treatment Process

In the dip coating and heat treatment process, the substrate is initially sandblasted to create a roughened surface, enhancing adhesion. FGC powder is then mixed into a slurry and manually sprayed onto the substrate. The coated substrate is subsequently dried in an oven under ambient conditions, followed by a fusion step at 900 °C [234]. This method is straightforward and well suited for small-batch production. By carefully adjusting the crystallization parameters, coatings with varied microstructures and properties can be obtained. The resulting coatings typically exhibit smooth surfaces, although they are prone to high brittleness and fragility. Furthermore, ensuring uniform coating thickness is challenging, making this method less ideal for applications involving substrates with complex geometries. Nevertheless, this approach is commonly employed for the preparation of coatings on restorative materials such as crowns and bridges.

#### 7.2.2. Magnetron Sputtering Technology

The magnetron sputtering process begins with the cleaning and pre-treatment of the substrate surface to enhance coating adhesion. FGC powder is then placed as the target material in a vacuum chamber filled with argon gas. Under the influence of a high-frequency electric field, the argon gas is ionized and accelerated, striking the target material and causing the atoms on the target surface to be ejected and deposited onto the substrate, forming a coating. The coated substrate is subsequently subjected to heat treatment to improve further the structure and properties of the FGC layer [57]. The coatings produced by magnetron sputtering offer excellent adhesion and enable precise control over coating thickness. However, this technique is characterized by a slow deposition rate and process complexity. It is particularly suited for the preparation of coatings on implant fixation bolts.

#### 7.2.3. CoBlast Process

The CoBlast process is a room-temperature coating technique wherein FGC powder is mixed with a spray medium and projected onto a rotating substrate via a single nozzle. The substrate is rotated at a controlled speed for a specified duration, and the particles rapidly melt and solidify upon contact with the substrate due to the high-pressure airflow [245]. This process addresses the limitations of high-temperature coating techniques and is capable of coating substrates with varying geometries and preparing composite coatings. However, it requires precise control over process parameters and specialized equipment, making it more complex and costly than other methods. As such, the CoBlast process is primarily applied in the preparation of coatings for dental implants.

#### 7.2.4. Suspension Plasma Process

The suspension plasma process involves mixing FGC powder, water, and dispersant in a specific ratio. The substrate is pre-treated through sandblasting and preheating to enhance bonding strength. The prepared suspension is then injected into an ion jet via a plasma spraying system, where it is subsequently sprayed onto the substrate [237]. Conventional plasma spraying typically requires powders with high particle size and fluidity, which are often achieved through additional processes such as grinding and spray granulation. In contrast, the suspension plasma process allows for the preparation of high-performance coatings with fine microstructures under the precise control of the process parameters. These coatings contribute to improved cellular responses, including reduced apoptosis and enhanced cell proliferation. Moreover, the lower deposition temperature in this process minimizes substrate damage. However, the determination of optimal process parameters can be challenging due to the multitude of influencing factors. Despite these complexities, the suspension plasma process is widely used in the fabrication of coatings for implants and dental restorations.

#### 7.2.5. Electrophoretic Deposition Technology

Electrophoretic deposition (EPD) involves dispersing FGC powder in ionized water or organic solvents to form a stable suspension. The substrate is then immersed in this suspension while an electric field is applied, causing the FGC powder to gradually deposit onto the substrate [240]. This method produces coatings with good uniformity and is particularly suitable for substrates with complex geometries. However, the coating thickness achieved through EPD is often limited, and the process can be prone to durability issues. Additionally, the procedure itself is relatively complex. Despite these limitations, EPD is commonly employed for coating dental implants and restorative materials due to its ability to achieve fine coatings on intricate shapes.

#### 7.2.6. FGC Particle Abrasion and Laser Fusion

This technique is typically applied in minimally invasive restorative procedures within the oral cavity. It begins with air particle abrasion, using silica-coated Al_2_O_3_ particles under pressure, followed by the application of FGC powder under similar conditions. The FGC powder is then sprayed onto the substrate’s surface, which is subsequently irradiated with a laser at specific parameters to form a bonded coating [243]. This method results in dense, strong coatings; however, it requires a high level of operator expertise, as improper handling can lead to suboptimal coating properties that may not meet restorative requirements. This technique is primarily used for the preparation of coatings for dental crowns, veneers, and other restorative materials.

FGC coatings offer superior degradation resistance when compared to hydroxyapatite coatings. Additionally, these coatings exhibit enhanced biocompatibility and antimicrobial properties relative to other coatings, such as Si_3_N_4_ and SiC. As a result, FGC coatings show great promise for applications in dental and orthopedic implants.

## 8. Challenges of Using FGC in Dentistry

Although FGC has garnered significant attention as a promising material for dental applications, several limitations and challenges persist.

### 8.1. Synthesis Techniques

Despite the availability of various synthesis methods for FGC, there remains considerable variability in the properties and purity of the resulting products, which complicates their industrial-scale production. One of the key challenges is the difficulty in producing high-purity materials in large quantities. While the melt-quenching technique is the most commonly employed method for large-scale production, it is associated with substantial energy consumption. Furthermore, although Zhu et al. successfully synthesized FGC at low temperatures by adding KF, MgF_2_, and CaF_2_, the mechanical properties of these low-temperature synthesized products are still inferior to those produced at higher temperatures [246]. Other synthesis methods, such as the sol–gel process, chemical precipitation, and mineralization techniques, face additional challenges, including complex synthesis protocols, long reaction times, high raw material costs, low purity, and insufficient strength of the resulting FGC. These limitations hinder the practical application of these materials on an industrial scale.

### 8.2. Material Properties

Another critical issue lies in the lack of systematic and comprehensive studies on the relationships between macro-level variables, microstructural features, second-phase formations, and the forming process, and their collective effects on key material properties such as biocompatibility, bacteriostasis, degradation resistance, aesthetic properties, abrasion resistance, and mechanical strength. Achieving an optimal balance among these properties is essential for the material’s clinical and industrial viability. For instance, increasing the sintering temperature can promote the growth of fluorapatite crystals, thereby improving the mechanical properties and wear resistance of the glass-ceramics. However, excessively long crystal grains may adversely affect the material’s biocompatibility. Additionally, the rate of F^−^ and Ca^2+^ release plays a critical role in the bacteriostatic properties and degradation resistance of the materials; however, the regulation and study of ion release rates remain insufficiently explored. Mechanical properties, particularly fracture toughness and flexural strength, are the key factors limiting the widespread application of FGC. Although various improvements have been made by optimizing the forming techniques, adjusting the composition of the synthetic materials, and incorporating reinforcing phases, defects such as porosity and cracking continue to impede further enhancements in material performance. Therefore, a deeper understanding of defect characterization, the mechanisms underlying defect formation, and the distribution patterns of these defects are crucial for advancing the mechanical properties and overall performance of FGC.

### 8.3. Forming Process

The preparation of fluorapatite dental restorations through traditional methods, such as the lost wax technique, pressureless sintering, hot pressing, CAD/CAM technology, and shell sintering, faces several significant challenges. These include limited flexibility in the forming process, difficulty in accommodating a wide variety of restoration shapes, and a resulting increase in production costs and inefficiencies. Additionally, these methods often lead to restorations with lower precision and suboptimal performance. While 3D printing technology offers promising potential for the preparation of FGC dental restorations, its application is hindered by several factors, including the complexity of post-processing, the lack of specialized AM equipment, and the unsuitability of certain small or intricate restorations for mass production. The absence of targeted equipment also leads to significant material wastage.

### 8.4. Application in Veneers

FGC has been explored as veneer materials; however, despite numerous efforts by researchers to address issues such as poor interfacial adhesion, mismatch in thermal expansion coefficients with the substrate, and stability concerns including aging, fatigue, and susceptibility to delamination and surface breakage, the manufacturing process remains complex. Additionally, the material and time costs associated with these glass-ceramics are still relatively high.

### 8.5. Applications in Composites

Although composites extend the performance of FGC and expand their applications, defects such as porosity are still a bottleneck for the further improvement of composites’ performance and a challenge for their application in dentistry. How to reduce the defects of restorations and further improve the performance of composites has not been thoroughly investigated.

### 8.6. Application in Coating

When employed as a coating material, FGC is prone to cracking due to a mismatch in the coefficient of thermal expansion between the coating and the substrate. This thermal expansion mismatch results in reduced adhesion at the interface, ultimately leading to the degradation of the coating. Moreover, the processes involved in preparing these coatings are complex, varied, and often difficult to control, which contributes to high processing costs and challenges in achieving consistent quality.

## 9. Future Perspectives

### 9.1. Synthesis Techniques

In terms of preparation methods, melt-quenching technology remains the dominant approach due to its large-scale synthesis capacity, excellent mechanical properties, and broad applicability. In contrast, alternative methods such as sol–gel, chemical precipitation, and mineralization have not been widely adopted in industrial production due to their high costs and low yields. Future advancements may focus on optimizing the existing synthesis processes and developing novel techniques to improve efficiency and reduce production costs.

### 9.2. Material Properties

FGC exhibits favorable aesthetic properties, as their mineral composition closely resembles that of natural teeth. They also demonstrate the ability to release F^−^ and Ca^2+^, which help regulate osmotic pressure and contribute to their bacteriostatic properties and biocompatibility. However, their relatively lower mechanical properties remain a significant limitation for broader clinical applications. To address this, biomimetic and bio-inspired composites, such as those mimicking pearl-like structures, are promising avenues for enhancing material performance. Additionally, the development of advanced multi-material 3D printing technologies could enable the fabrication of more durable and aesthetically pleasing composites. Introducing the dislocation mechanism, typically observed in metals, into FGC could further strengthen their mechanical properties, representing a viable direction for future research.

### 9.3. Forming Process

For the fabrication of dental restorations with diverse types and morphological features, advanced techniques such as lost wax casting, pressureless sintering, hot press sintering, and CAD/CAM technologies are commonly employed. Notably, shell sintering technology has opened new possibilities for the preparation of FGC dental restorations. The potential of 3D printing technology in this domain is substantial, and the optimization of key processes—including paste composition, printing parameters, degreasing, and sintering—will be critical to enhancing the application of 3D printing for FGC restorations. Furthermore, the advent of 4D printing technology, which introduces the element of time as a fourth dimension, offers the ability to design restorations that respond to external stimuli and undergo morphological changes. This innovative approach may allow dental restorations to self-regulate in the oral environment, thereby further optimizing their structure and improving their bioactivity [247].

### 9.4. Application in Veneers

FGC, when used as veneer materials, are susceptible to issues such as veneer porcelain delamination and fragmentation. The integration of CAD/CAM technology for the fabrication of veneer ceramics has been shown to provide superior bond strength compared to traditional manual layering, pressing, and cementing techniques. Various surface modification approaches, including the use of connectors, gaskets, washing layers, sandblasting, surface polishing, meshing, and laser or acid etching, are commonly employed to enhance the bond strength between zirconia and FGC bilayer ceramics. Moreover, the application of 3D printing technology to create complex interlocking mating structures in constrained spaces represents a promising solution to address the problem of bilayer ceramic delamination. In parallel, the development of adhesives with enhanced bonding capabilities is crucial for improving the adhesion of veneered ceramics to the underlying core material.

### 9.5. Applications in Composites

The use of composites is an effective strategy to enhance the mechanical properties of FGC and expand their range of applications. However, in comparison to ZrO_2_, Al_2_O_3_, and LD glass-ceramic restorations, the mechanical properties of both FGC and their composite restorations remain relatively low. Defects such as porosity are a major limiting factor in the mechanical performance of these composites. A thorough understanding of defect distribution, formation mechanisms, and strategies to reduce these defects is essential for improving the mechanical properties of composite materials. For instance, industrial computed tomography technology can be employed to characterize defects, while microwave body-center heating can alter the heating mode to improve densification during fabrication. Additionally, for 3D-molded restorations, the selection of appropriate resins, dispersants, and other additives, along with the optimization of the degreasing and sintering processes, is critical to enhancing the mechanical properties of the composites.

### 9.6. Application in Coating

FGC coatings are highly effective in enhancing bone resorption in implants and other biomedical applications due to their excellent degradation resistance, corrosion resistance, and biocompatibility. The preparation techniques used for these coatings are crucial to their quality and performance. Methods such as dip coating with subsequent heat treatment, magnetron sputtering, CoBlast processing, suspension plasma techniques, electrophoretic deposition, and FGC particle abrasion followed by laser melting each offer distinct advantages and drawbacks. The selection of the most appropriate method depends on the specific application requirements and conditions. Future research should focus on optimizing process parameters, improving coating uniformity and adhesion, and reducing production costs to facilitate the broader application of FGC coatings in dental and biomedical fields.

## 10. Conclusions

This paper provides a comprehensive review of FGC’s research progress and applications in dental restorations, encompassing their synthesis techniques, material properties, forming methods, and applications in veneers, composites, and coatings. Additionally, the challenges and future development prospects of FGC in dental applications are discussed. As dental technology continues to advance, the innovative manufacturing of FGC remains critical. The aim is to overcome the existing challenges of inefficient synthesis, performance limitations, and complex molding processes, with a view to ultimately promoting the wider use of the material in dentistry and better meeting the needs of patients in treatment.

## Figures and Tables

**Table 1:** Composition and content of raw materials for the preparation of FGC by melt-quenching.

Composition	Proportion and Range of Each Component						
	wt%				mol%		
SiO_2_	54.6	54.6	9.5	40	31.5–33.9	4.5–7.5	28.4–38.1
Al_2_O_3_	14.2	14.4	3	-	0.9–10.4	3–5	-
MgO	-	-	7.2	-	21.2–22.9	-	-
Nb_2_O_5_	-	-	-	-	0.2–0.3	-	-
Na_2_O	8.4	8.6	-	21	2.3–2.5	-	-
K_2_O	10.7	4.2	0.93	-	3.8–4.1	-	-
ZnO	3	3	-	-	-	-	-
P_2_O_5_	4	4	31.7	15	5–5.4	0–1.5	4.7–6.3
F	0.7	0.7	-	-	-		-
CaO	5	6	37.5	12	8.6–23.9	0–5	41.1–55.5
CaF_2_	-	-	5.8	16.71	11.2–12	0–2	0–25.5
ZrO_2_	0.9	1.5	-	-	-	-	-
TiO_2_	0.2	1	-	-	-	-	-
Li_2_O	0.2	0.2	-	-	-	-	-
B_2_O_3_	0.3	1	-	-	-	-	-
CeO_2_	0.8	0.8	-	-	-	-	-
K_2_SiF_6_	-	-	4.4	-	-	-	-
reference	[21,22,23]	[24,25]	[26]	[27]	[11]	[28,29,30,31]	[32]

**Table 2:** Comparison of mechanical properties of FGC synthesis methods.

Synthetic Method	Flexural Strength (MPa)	Fracture Toughness (MPa·m^1/2^)	Elastic Modulus (GPa)	Hardness (Hv/GPa)	Reference
Melt-Quenching	40–160	-	67–95	490–650 Hv	[21,22,23,24,25,26,27]
Sol–Gel	27	-	20	7.7 GPa	[33]
Chemical Precipitation	-	1.7–2	90–100	97–106 GPa	[39,40]
Mineralization Method	-	1.3	-	4.1 GPa	[43]

**Table 3:** Material selection table for clinical application.

Type	Applicable Characteristics	Material Selection Criteria	Clinical Outcome
Tooth repair	Biocompatibility and wear resistance	Good aesthetic properties and wear resistance are required.	Improve the function after repair, reduce infection, and the long-term effect is good
Pulp canal therapy	Biocompatibility and sealing performance	It must have good sealing and antibacterial properties.	Improve the therapeutic effect and reduce postoperative complications
Implant coatings	Biocompatibility	Long-term stability is required to reduce rejection.	Enhance the stability of the implant and reduce the failure rate.

**Table 4:** In vitro friction and wear test results for FGC and their composites.

Material	Forming Method	Antagonist	Load	Frequency or Speed	Radius or Stroke	Time	Lubricant	The Volume, Rate, or Height of Wear	Author
FGC	pressing	Orthodontic extracted teeth	20–60 N	50–250 r/min	Radius of rotation 6 mm	-	The saliva of volunteers	0.01–1 mm^3^	[105]
FGC	pressing	Orthodontic extracted teeth	40 N	150 r/min	Radius of rotation 6 mm	10 min	A mixture of corn powder and distilled water	0.01–1.2 mm^3^	[106]
FGC	pressing	Steatite	35 N	2 Hz	Radius of rotation 1.5 mm	90 min	Artificial saliva	0.32–0.56 (×10^−3^ mm^3^/N·m)	[24]
FGC	pressing and ion exchange strengthening	Yttria stabilized zirconia	10 N	200 r/min	-	64–128 h	Artificial saliva	Height 20–38 μm	[111]
FGC	SLA 3D printing	TA2 balls	20 N	2 Hz	9 mm	60 min	Artificial saliva	0.6–1.7 mm^3^	[112]
Composite of FGC and Al_2_O_3_ whiskers	SLA 3D printing	TA2 balls	20 N	2 Hz	9 mm	60 min	Artificial saliva	2.4–16.2 (×10^−5^ mm^3^/N⋅m)	[114]
Composites of FGC and ZrO_2_ fiber	SLA 3D printing	TA2 balls	20 N	2 Hz	9 mm	60 min	Artificial saliva	5.2–21.3 (×10^−5^ mm^3^/N⋅m)	[115]

**Table 5:** Standard requirements for mechanical properties of common dental restorations.

Type of Restoration	Type of Performance	Back-End Unit Crown	Veneers	Inlays/Onlays	Front End 3 Unit Bridge	Posterior Teeth or More Than Four Units of the Bridge
performance requirement	flexural strength	≥100 MPa	≥50 MPa		≥300 MPa	≥800 MPa
	fracture toughness	≥1 MPa·m^1/2^	≥0.7 MPa·m^1/2^		≥2 MPa·m^1/2^	≥5.0 MPa·m^1/2^

**Table 6:** Comparative study of mechanical properties of FGC and their composites with other types of dental materials.

Material	Flexural Strength (MPa)	Fracture Toughness (MPa·m^1/2^)	Elastic Modulus (GPa)	Hardness (Hv/GPa)	Translucency Parameters	Forming Method	Reference
FGC	160	-	87	637 Hv	-	Pressing	[106]
FGC	165	-	95	650 Hv	15–25.3	Pressing	[24,125,126]
FGC	205.97	-	97.06	772.05 Hv	-	SLA	[112]
FGC/ZrO_2_ particles	240.80	3.49	100	620 Hv	-	SLA	[127]
FGC/Al_2_O_3_ whiskers	220.3	1.8	107	610 Hv	-	SLA	[114]
ZrO_2_	300–1200	5–10	210	5–15 GPa	5.5–15.1	SLA/DLP	[128,129,130,131,132,133,134]
Al_2_O_3_	400–600	3.5–4	380	15–22 GPa	5.5–15.1	SLA/DLP	[135,136,137,138,139,140,141,142,143]
LD	250–500	2–3.5	90–100	5–8.1 GPa	12–23	SLA/DLP	[144,145,146]
Glass-ceramic(leucite,mica)	55–160	0.6–2.2	48–164	3.2–7.9 GPa		Pressing	[147,148,149,150]
